# Supplementary material for: Bio-Inspired Nanostructured Ti-6Al-4V Alloy: The Role of Two Alkaline Etchants and the Hydrothermal Processing Duration on Antibacterial Activity
Source: Nanomaterials (Basel). 2022 Mar 29;12(7):1140. doi: 10.3390/nano12071140 (PMC9000892; doi:10.3390/nano12071140)
Supplement: Supplementary file 1 [file nanomaterials-12-01140-s001.zip › nanomaterials-1637272-supplementary.pdf]

nanomaterials

Supplementary Materials

Bio-Inspired Nanostructured Ti-6Al-4V Alloy: The Role of Two Alkaline Etchants and the Hydrothermal Processing Duration on Antibacterial Activity

Richard Bright [1], Andrew Hayles [1], Jonathan Wood [1], Neethu Ninan [1], Dennis Palms [1], Rahul M. Visalakshan [2], Anouck Burzava [1], Toby Brown [3], Dan Barker [3] and Krasimir Vasilev [1,4,*]

[1] Academic Unit of STEM, University of South Australia, Mawson Lakes, Adelaide, SA 5095, Australia; richard.bright@unisa.edu.au (R.B.); andrew.hayles@unisa.edu.au (A.H.); jonathan.wood@mymail.unisa.edu.au (J.W.); neethun.ninan@gmail.com (N.N.); dennis.palms@unisa.edu.au (D.P.); anouck.burzava@unisa.edu.au (A.B.)
[2] Division of Biomaterials and Biomechanics, Department of Restorative Dentistry, School of Dentistry, Oregon Health and Science University, Portland, OR 97201, USA; madathip@ohsu.edu
[3] Corin Australia, Baulkham Hills, NSW 2153, Australia; tobydbrown@gmail.com (T.B.); dan.barker@coringroup.com (D.B.)
[4] College of Medicine and Public Health, Flinders University, Bedford Park, SA 5042, Australia
* Correspondence: krasimir.vasilev@unisa.edu.au

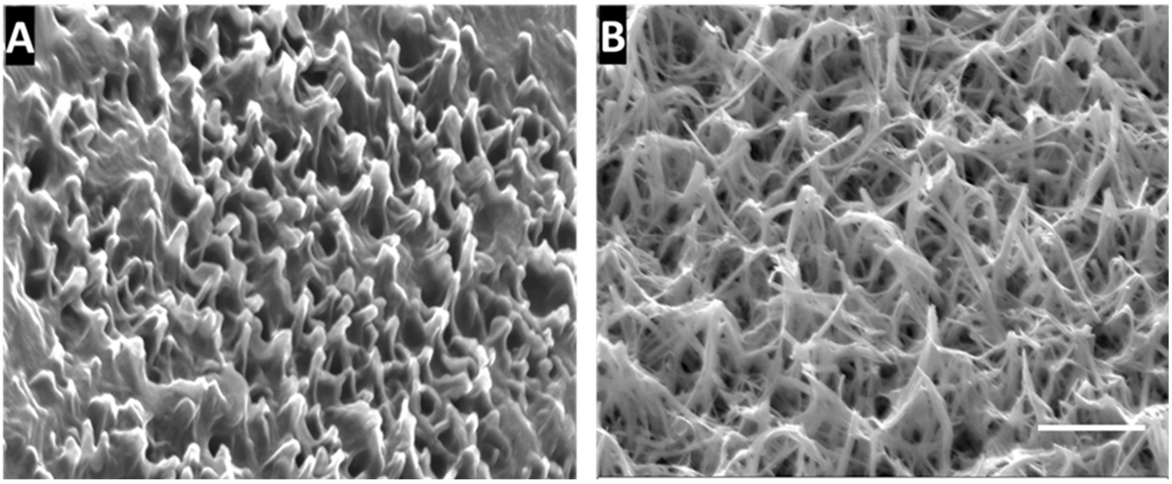

Figure S1. SEM micrographs comparing nanostructures from a dragonfly wing (**A**) and hydrothermally etched nanostructures (**B**). Scale bar represents 500 nm.

Table S1. Geometry and distribution of the nanospikes prepared on the surface of Ti6Al4V by KOH and NaOH-aqueous etching solutions over different time periods. The values were presented in means ± standard deviation from at least 20 spikes by analyzing at least four fields of view. Spacing distances were calculated from the SEM micrographs with the stage at orthogonal position. Highlighted measures represent the best antibacterial activity for the two alkaline etching solutions (NaOH and KOH).

**Etching Solution**	**NaOH**				**KOH**			
**Etching Time:**	**1 h**	**3 h**	**4 h**	**5 h**	**1 h**	**3 h**	**4 h**	**5 h**
Height (nm)	112 ± 27	185 ± 38	367 ± 80	425 ± 107	122 ± 107	242 ± 73	207 ± 37	340 ± 175
Diameter at mid-height (nm)	30 ± 4	94 ± 33	62 ± 23	83 ± 30	65 ± 10	71 ± 11	85 ± 18	83 ± 32
Spacing (nm)	185 ± 41	190 ± 42	182 ± 48	224 ± 55	330 ± 83	453 ± 122	500 ± 120	544 ± 150
Density(spike/μm²)	42 ± 4	35 ± 7	75 ± 8	32 ± 9	17 ± 6	10 ± 2	10 ± 3	8 ± 2

Table S2. AFM roughness measurements and calculated surface area. RMS = root mean square, R_a = average roughness and SA = calculated surface area.

**Treatment**	**RMS (nm)**	**R_a (nm)**	**SA (μm²)**
AR-Ti	10.1	6.6	25.2
NaOH-4h	88.4	71.4	79.9
KOH-5h	88.5	61.5	50.3

Table S3. Mean difference, confidence intervals and *p*-values determined using Tukey's multiple comparisons test for HDF cell viability.

**Treatment**	**Mean Diff.**	**95.00% CI of diff.**	**Significance**	**P Value**
AR-Ti vs. AMNa4h	−2.505	−38.33 to 33.32	ns	0.975
AR-Ti vs. AMK5h	−5.47	−41.29 to 30.35	ns	0.8882
AMNa4h vs. AMK5h	−2.965	−38.79 to 32.86	ns	0.9653
